# Antibiotic perturbation of mixed-strain *Pseudomonas aeruginosa* infection in patients with cystic fibrosis

**DOI:** 10.1186/s12890-017-0482-7

**Published:** 2017-11-02

**Authors:** Anna S. Tai, Laura J. Sherrard, Timothy J. Kidd, Kay A. Ramsay, Cameron Buckley, Melanie Syrmis, Keith Grimwood, Scott C. Bell, David M. Whiley

**Affiliations:** 1 School of Medicine, The University of Queensland, Brisbane, QLD Australia; 2 Adult Cystic Fibrosis Centre, Department of Thoracic Medicine, The Prince Charles Hospital, Brisbane, QLD Australia; 3 Western Australia Adult Cystic Fibrosis Centre, Department of Respiratory Medicine, Sir Charles Gairdner Hospital, Hospital Avenue, Perth, WA 6009 Australia; 4 Lung Bacteria Group, QIMR Berghofer Medical Research Institute, Brisbane, QLD Australia; 5 School of Chemistry and Molecular Biosciences, The University of Queensland, Brisbane, QLD Australia; 6 Centre for Experimental Medicine, Queen’s University Belfast, Belfast, UK; 7 Child Health Research Centre, The University of Queensland, Brisbane, QLD Australia; 8 UQ Centre for Clinical Research, The University of Queensland, Brisbane, QLD Australia; 9 Pathology Queensland, Microbiology Department, Brisbane, QLD Australia; 10 Menzies Health Institute Queensland, Griffith University and Gold Coast Health, Gold Coast, QLD Australia

**Keywords:** Cross-infection, Cystic fibrosis, Disease progression, *Pseudomonas Aeruginosa*, Respiratory tract infections

## Abstract

**Background:**

Pulmonary exacerbations in cystic fibrosis (CF) remain poorly understood and treatment is usually targeted at *Pseudomonas aeruginosa*. Within Australia a predominant shared *P. aeruginosa* strain (AUST-02) is associated with greater treatment needs. This single centre study assessed temporal shared strain population dynamics during and after antibiotic treatment of exacerbations.

**Methods:**

Sputum was collected from 12 adult patients with a history of chronic AUST-02 infection at four time-points during and after treatment of an exacerbation. Forty-eight *P. aeruginosa* isolates within each sample underwent AUST-02 allele-specific PCR and SNP-based strain genotyping.

**Results:**

Various commonly shared Australian strains (AUST-01, 0.1%; AUST-02, 54.3%; AUST-06, 36.6%; AUST-07, 4.6%; AUST-11, 4.3%) and two unique strains (0.1%) were identified from 45 sputum samples (2160 isolates). Based on within-patient relative abundance of strains, a “single-strain infection” (*n* = 7) or “mixed-strain infection” (*n* = 5) was assigned to each patient. A significant temporal variation in the *P. aeruginosa* population composition was found for those with mixed-strain infection (*P* < 0.001). Patients with mixed-strain infections had more long-term treatment requirements than those with single-strain infection. Moreover, despite both groups having similar lung function at study entry, patients with single-strain infection had greater improvement in FEV_1_% predicted following their exacerbation treatment (*P* = 0.02).

**Conclusion:**

Pulmonary exacerbations may reveal multiple, unrelated *P. aeruginosa* strains whose relative abundance with one another may change rapidly, in a sustained and unpredictable manner.

**Electronic supplementary material:**

The online version of this article (10.1186/s12890-017-0482-7) contains supplementary material, which is available to authorized users.

## Background

Despite improved survival for people with cystic fibrosis (CF) [1], most still die prematurely from chronic pulmonary infections characterised by recurrent exacerbations, progressive lung function decline, increased treatment requirements and reduced quality of life [2–6]. The pathophysiology of pulmonary exacerbations is nevertheless poorly understood and a standardised definition of an exacerbation remains elusive [7–9].

*Pseudomonas aeruginosa* is the most common pulmonary pathogen in CF and antibiotic treatment of exacerbations directed against this organism is pivotal to patient management [10]. Once *P. aeruginosa* becomes established within the airways of patients with chronic lung disease, it is usually by a single strain that evolves through micro-adaptation into multiple sub-lineages of the original ancestral clone [11, 12]. There are however, reports of co-infection with two or more distinct *P. aeruginosa* genotypes in both CF [13–15] and, non-CF bronchiectasis [12]. In Australia, a predominant shared strain, AUST-02, was detected in 18% of all patients with *P. aeruginosa* infection nationally and was associated with increased centre visits and intravenous antibiotic courses [13]. Given the high-prevalence and clinical significance of the AUST-02 strain within Australian centres, including its predominance in our own clinic, the primary aim of this study was to assess AUST-02 population stability and determine whether other *P. aeruginosa* strains emerged during and after treating an exacerbation.

## Methods

### Study patients and characteristics

Twelve CF patients (aged ≥18-years) with chronic AUST-02 infection, defined according to the modified Leeds Criteria [16, 17], were recruited over a 5-month period between February and June 2014 following admission to The Prince Charles Hospital (Brisbane, Australia) for intravenous antibiotic treatment of an exacerbation (as defined by the Fuchs criteria [9]). While in hospital, CF patients with *P. aeruginosa* were not managed exclusively in single room accommodation with some admitted to shared rooms [18]. The Prince Charles Hospital Human and Research Ethics Committee, Metro North Hospital and Health Service, Brisbane, Queensland, Australia approved the study (HREC/13/QPCH/127) and all participants provided written, informed consent.

Baseline characteristics of age, gender, cystic fibrosis transmembrane conductance regulator (CFTR) genotype, and the best lung function (forced expiratory volume in the first second percentage predicted; FEV_1_% predicted [19]) and body mass index (BMI) recorded in the previous year were collected from hospital records. Years chronically infected by *P. aeruginosa*, clinical care requirements in the previous year (number of hospital admissions, inpatient days and outpatient clinic visits), types of treatments in the previous year (oral azithromycin, inhaled tobramycin, inhaled colistin) and diagnoses of diabetes and liver disease were also recorded. Clinical data collected during their hospitalisation included FEV_1_% predicted and BMI at the start-of-treatment and end-of-treatment time-points. Serum C-reactive protein (CRP) was measured on days 3 and 10 of inpatient stay, according to a local treatment protocol (for consistency hereafter referred to as start-of-treatment and end-of-treatment). Types of antibiotics administered, antibiotic duration, relapse (defined as readmission to hospital with a further pulmonary exacerbation before outpatient review occurred) and time-to-next hospitalisation for an exacerbation were collated.

### Sputum collection, culture and genotyping

Spontaneously expectorated sputum was collected at study entry when commencing intravenous antibiotics (‘start-of-treatment’), during the first-week of treatment (‘during treatment’), at its completion (‘end-of-treatment’) and during outpatient review following discharge or if readmission to hospital before outpatient review occurred (‘follow-up’).

Sputum samples were processed as described previously to isolate single colonies [20]. Quantitative cultures were performed using standard techniques. A sweep of colonies from the ‘start-of-treatment’ sputum samples was tested for AUST-02 by an allele-specific polymerase chain reaction (PCR; Additional file 1: Supplementary Methods). In addition, 48 individual presumptive *P. aeruginosa* colonies were selected randomly for genotyping from each sputum sample the 12 study patients provided.

**Fig. 1:**
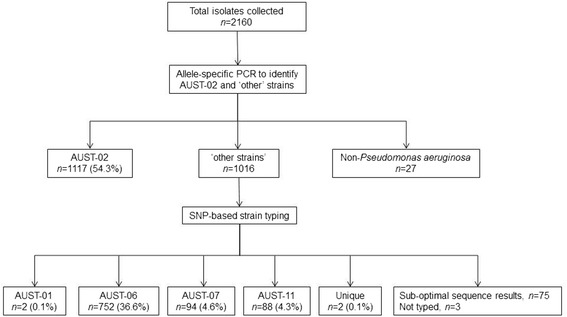
Number of isolates randomly selected from 45 sequential sputum samples provided by 12 patients with cystic fibrosis and results of subsequent strain identification by molecular typing methods. All isolates identified to the strain-level were included in the final analyses (*n* = 2055)

Bacterial DNA preparation was undertaken by heat-denaturation [21]. Each isolate underwent testing by the above AUST-02 allele-specific PCR (Fig. 1 and Additional file 1: Supplementary Methods) with isolates testing negative for AUST-02 subjected to single nucleotide polymorphism (SNP)-based strain typing (Agena; formerly Sequenom iPLEX) at the Australian Genome Research Facility (AGRF, The University of Queensland, Brisbane) based on a protocol described previously [22].

### Phenotypic testing

The first recruited subject had a mixed-strain infection and each *P. aeruginosa* isolate genotyped from this patient underwent additional phenotypic testing to determine the within-host diversity. This sub-analysis involved 186 isolates with a total of 2790 phenotypic tests completed. Susceptibility to 11 anti-pseudomonal antibiotics was determined by disc diffusion [23], auxotrophy by growth on M9 minimal media [11] and alginate overproduction by the appearance of mucoid isolates on Pseudomonas Isolation Agar after overnight aerobic incubation at 36 °C [24]. Inactivating *lasR* mutant colonies were identified by their metallic iridescent sheen on Luria Bertani agar following overnight incubation in aerobic conditions at 36 °C [25], while pyocyanin production was quantified by measuring the A_695_ value as described previously [11].

### Statistical analyses

A repeated measures analysis of variance (ANOVA) was used to examine if the log-transformed *P. aeruginosa* total viable count differed between time-points of sputum collection. Clinical variables were compared between time-points or groups using a paired t-test (or Wilcoxon signed-rank test), independent t-test (or Mann-Whitney test) or Fisher’s exact test, as appropriate. Variation in the number of strains detected and phenotypic traits were assessed by chi-square tests. All statistical analyses were generated using SPSS version 22. A two-tailed, *P* < 0.05 was considered statistically significant.

## Results

Baseline patient characteristics are summarised in Table 1 (individual data, Additional file 2: Table S1 and Additional file 3: Table S2). All patients had advanced lung disease, had been chronically infected with *P. aeruginosa* for several years, and with one exception were taking maintenance oral azithromycin with inhaled anti-pseudomonal antibiotics prior to the exacerbation episode. Between-group differences at baseline were not statistically significant. A limited numbers of bacterial co-pathogens were isolated from sputum samples patients at baseline and within prior 3 months (4 patients with methicillin-susceptible *Staph. aureus*), During the period of exacerbation treatment, all patients received combination intravenous anti-pseudomonal antibiotics and continued taking azithromycin. Inhaled antibiotics were ceased. The median (range) period from completion of intravenous antibiotics and follow-up visit was 42 (13 – 119) days.

**Table 1:** Characteristics of the 12 patients with single-strain and mixed-strain infections

Variable	Single-strain infection (*n* = 7)	Mixed-strain infection (*n* = 5)	All ^a^ (*n* = 12)
Age, mean years (SD)	33.1 (11.2)	30.4 (6.1)	32.0 (9.2)
Gender, Male, *number* (%)	5 (71.4)	4 (80.0)	9 (75.0)
P.Phe508del heterozygous, *number* (%)	5 (71.4)	1 (20.0)	6 (50.0)
P.Phe508del homozygous, *number* (%)	2 (28.6)	4 (80.0)	6 (50.0)
FEV_1_% predicted, mean (SD)	45.9 (20.8)	45.6 (15.3)	45.8 (17.9)
BMI, mean kg/m^2^ (SD)	23.7 (6.7)	21.6 (3.3)	22.9 (5.4)
Chronic *Pseudomonas aeruginosa* infection >10-years, *number* (%)	6 (85.7)	5 (100)	11 (91.7)
Clinical care in previous year, mean (SD)			
Number of hospital admissions	2.1 (2.0)	4.8 (2.9)	3.3 (2.7)
Number of inpatient days	29.1 (30.0)	69.0 (37.1)	45.8 (37.6)
Number of outpatient clinic visits	11.6 (8.7)	16.2 (3.0)	13.6 (7.1)
Regular treatments prescribed in previous year, number (%)			
Oral azithromycin	7 (100)	5 (100)	12 (100)
Inhaled colistin	3 (42.9)	3 (60.0)	6 (50.0)
Inhaled tobramycin	6 (85.7)	4 (80.0)	10 (83.3)
Diabetes, *number* (%)	1 (14.3)	1 (20.0)	2 (16.7)

*Abbreviations*: *BMI* body mass index, *FEV1%* predicted, forced expiratory volume in the first second percentage predicted; *SD* standard deviation

^a^No patients were diagnosed with CF-related liver disease

### *Pseudomonas aeruginosa* total viable count

**Fig. 2:**
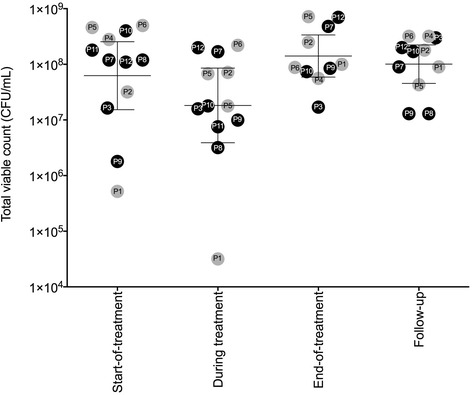
Geometric means (with 95% confidence intervals) of total viable counts expressed as colony-forming units (CFU)/mL of *Pseudomonas aeruginosa* in sputum samples collected from adult patients with cystic fibrosis at start-of-treatment, during treatment and end-of-treatment time-points for intravenous antibiotic therapy of a pulmonary exacerbation, and at follow-up. Single-strain infection, black circle; mixed-strain infection, grey circle. There was no statistically significant difference between time-points of sputum collection and total viable counts (*P* = 0.1, repeated measures ANOVA; based on log-transformed CFU/mL from 10 patients, who each provided four sputum samples). There was also no statistically significant difference between time-points of sputum collection and total viable counts of those with single-strain infections (*P* = 0.5, repeated measures ANOVA; based on log-transformed CFU/mL from 5 patients, who each provided four sputum samples) or mixed-strain infections (*P* = 0.2, repeated measures ANOVA; based on log-transformed CFU/mL from 5 patients)

For all patients, the geometric mean *P. aeruginosa* total viable count was similar across the four consecutive time-points of sputum collection (start-of-treatment, 6.3 × 10^7^ colony-forming units (CFU)/mL; during treatment, 1.8 × 10^7^ CFU/mL; end-of-treatment, 1.4 × 10^8^ CFU/mL; follow-up, 1.0 × 10^8^ CFU/mL) with no statistically significant change over time (*P* = 0.1, repeated measures ANOVA; Fig. 2).

### Genotyping

**Fig. 3:**
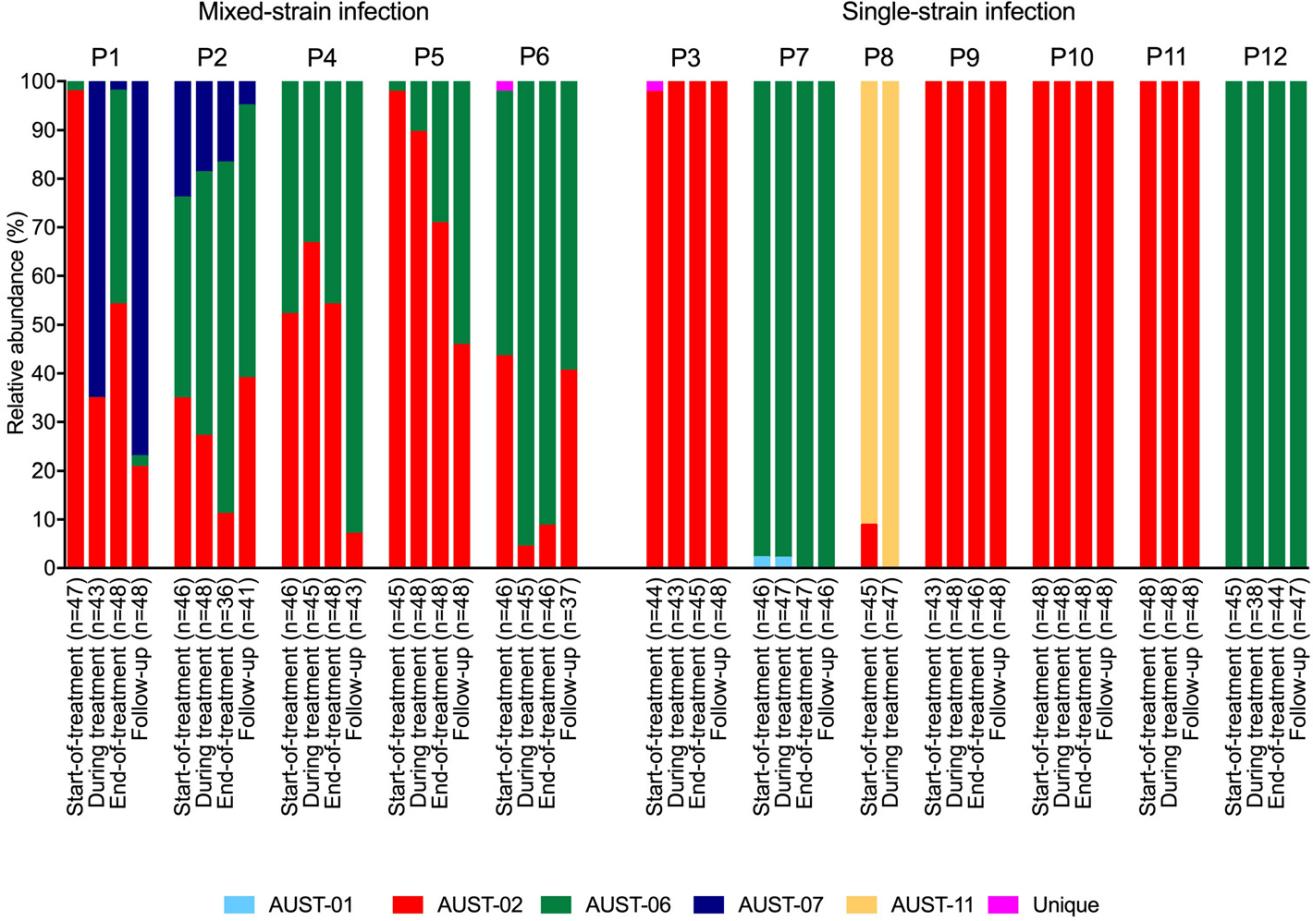
Relative abundance of *Pseudomonas aeruginosa* strains from the 12 patients with cystic fibrosis during intravenous antibiotic treatment of a pulmonary exacerbation and subsequent follow-up. End-of-treatment and/or follow-up sputum samples were unable to be provided by patients P8 and P11

The 12 study participants provided 45 sputum samples (Additional file 4: Figure S1), from which 2160 isolates were selected for genotyping (Fig. 1). Of the 2055 isolates genotyped successfully, 1117 (54.4%) were AUST-02 strains, while the remaining isolates comprised other commonly shared Australian strains (AUST-01, *n* = 2, 0.1%; AUST-06, *n* = 752, 36.6%; AUST-07, *n* = 94, 4.6%; AUST-11, *n* = 88, 4.3%) [13, 22], with only two isolates (0.1%) representing unique strains (Fig. 1). Although allele-specific PCR of a sweep of colonies confirmed all 12 patients harboured AUST-02 at study entry, this strain was not detected amongst any of the individual isolates selected from two patients at each of the four separate time-points; one patient (P12) was positive for AUST-06 only and another (P7) for AUST-01 and AUST-06 (Fig. 3).

### Mixed-strain dynamics

Based on relative abundances of *P. aeruginosa* strains in each sample, patients were categorised into two groups: those with a (1) “single strain infection” (P3, P7, P8, P9, P10, P11, P12) since a single strain comprised >90% relative abundance in all samples tested, and (2) “mixed-strain infection” (P1, P2, P4, P5, P6) where various mixtures of strains (at <90% relative abundance) were observed (Fig. 3).

A significant temporal variation in the *P. aeruginosa* population composition was found for the five patients with mixed-strain infections (*χ* ^*2*^ = 56.3, *P* < 0.001). A gradual reduction in the mean relative abundance (95% confidence interval) of AUST-02 was detected in sequential sputum samples compared to the start-of-treatment sample [during treatment, −20.7% (−58.4 to 17.0%); end-of-treatment, −25.4% (−46.7 to −4.1%); follow-up, −34.6% (−77.2 to 8.1%)]. Concurrently, the increased relative abundance of ‘other’ strains detected compared to the start-of-treatment was primarily due to a surge in AUST-06 richness at all time-points [during treatment, +9.1% (−16.7 to 34.9%); end-of-treatment, +26.9% (5.7 to 48.1%); follow-up, +23.4% (−6.0 to 52.8%)]. AUST-02 and AUST-06 temporal dynamics are summarised in Additional file 6: Figure S2.

### Phenotypic traits

Overall, 186 isolates from patient P1 (6/192 isolates were excluded as they were not genotyped successfully) underwent in-vitro assessment of adaptive phenotypic traits (Additional file 5: Table S3). A statistical association was detected between strain (AUST-02, AUST-06, AUST-07) and susceptibility category for 7/11 antibiotics with an equal or higher proportion of AUST-02 isolates categorised as non-susceptible to these seven antibiotics compared to AUST-06 or AUST-07 (*χ* ^*2*^ [range] = 26.0-108.9; *P* < 0.001, Additional file 7: Figure S3a). AUST-02 was also more often auxotrophic (92% isolates) compared to AUST-06 (35% isolates) and AUST-07 (2% isolates) (*χ* ^*2*^ = 131.7; *P* < 0.001, Additional file 7: Figure S3b). Defining phenotypic traits of AUST-06 and AUST-07 were mucoidy (87% of isolates; *χ* ^*2*^ = 134.9; *P* < 0.001, Additional file 7: Figure S3b) and colony surface iridescent sheen (86% of isolates; *χ* ^*2*^ = 108.0; *P* < 0.001, Additional file 7: Figure S3b), respectively. Pyocyanin production was similar between all strains (Additional file 7: Figure S3b).

### Clinical response to antibiotic treatment

Despite similar baseline FEV_1_% values and other between-group variables not being significantly different, patients harbouring mixed strains had more long-term treatment requirements than those with single strain infection (Table 1). During treatment of the exacerbation, there was a greater improvement in FEV_1_% predicted in patients with single-strain infection [median change (range): 7.2% (3.1 to 15.8%); *P* = 0.02; Wilcoxon signed-rank test; Table 2a] compared to those with a mixed-strain infection [median change (range): 2.4% (0 to 14.5%); *P* = 0.07; Wilcoxon signed-rank test; Table 2a), despite the median FEV_1_% predicted being similar between both groups at start-of-treatment [single-strain infection: 31.2% (range: 20.2 to 84.0%); mixed-strain infection: 41.7% (Range: 30.3 to 63.1%); *P* = 0.5, Mann-Whitney U test]. Furthermore, mean CRP was higher in those with single-strain (33.2 mmol/L) compared to mixed-strain infection (10.3 mmol/L) at start-of-treatment, albeit not significantly (*P* = 0.08, Welch’s t-test). After their exacerbation was treated, patients with mixed-strain infections had a shorter time to next admission for exacerbation treatment compared to those with single-strains, but this also did not reach statistical significance (Table 2b).

**Table 2:** Comparison of clinical outcome data for the 12 patients with single- and mixed-strain infections

Clinical parameter	Single-strain infection					Mixed-strain infection				
	Patients (number)	Start-of-treatment Mean (SD)	End-of-treatment Mean (SD)	Mean difference (95% CI)	*P*-value	Patients (number)	Start-of-treatment Mean (SD)	End-of-treatment Mean (SD)	Mean difference (95% CI)	*P*-value
(a) Response to treatment										
FEV_1_% pred ^a^	7	31.2 (20.2 to 84.0)	36.0 (31.2-99.8)	7.2 (3.1 to 15.8) ^b^	0.02	5	41.7 (30.3 to 63.1)	41.6 (30.3 to 65.5)	2.4 (0.0 to 14.5) ^b^	0.07
BMI (kg/m^2^)	7	23.2 (6.4)	23.3 (6.3)	0.1 (−0.3 to 0.6)	0.51	5	21.6 (3.5)	21.8 (3.9)	0.2 (−0.9 to 1.2)	0.65
CRP (mmol/L)	7	32.2 (28.6)	6.2 (4.9)	−27.0 (−53.9 to −0.2)	0.05	5	10.3 (8.7)	11.1 (8.0)	0.7 (−2.2 to 3.6)	0.52
Treatment length; mean days (SD)				15.0 (4.3)					16.6 (6.6)	0.65
(b) Longer-term outcomes										
	Single-strain infection (*n* = 7)					Mixed-strain infection (*n* = 5)				*P*-value
Relapse ^c^; no. (%)	0 (0)					2.0 (40.0)				0.15
Hospitalisation recurrence ^d^; median days (range)	154.0 (39.0 to 711.0)					85.0 (44.0 to 145.0)				0.12

*Abbreviations*: *BMI* Body mass index, *CI* confidence interval, *CRP* C-reactive protein, *FEV*_*1*_ *% pred* forced expiratory volume in the first second percentage predicted, *SD* standard deviation

^a^median (range) shown for all FEV1% pred data

^b^Wilcoxon signed rank test used to assess within-pair differences

^c^Defined as readmission to hospital before outpatient review occurred

^d^Time to next recurrence requiring hospitalisation; data censored for one patient with single-strain infection (time to end of data collection as no recurrence of hospitalisation experienced)

## Discussion

Shared *P. aeruginosa* strain dynamics during and after antibiotic treatment of a pulmonary exacerbation in chronically infected CF patients were investigated by combining in-depth culture and genotyping techniques. Interestingly, this Australian study found that a substantial number of CF patients showed mixed-strain infection, which contrasts with the extensive experience in the UK where similar studies of the Liverpool Epidemic Strain (LES) consistently demonstrated that CF patients have chronic infection with a single strain, which has diverged into multiple co-existing sublineages in the airways [11, 26]. This disparity is possibly related to differences in patient segregation practices between study centres.

Patients with mixed-strain infection showed significant changes in strain composition throughout the entire exacerbation episode and follow-up period, with others typically maintaining the same strain. Almost all other non-AUST-02 isolates (>99%) identified comprised previously recognised Australian shared strains, suggesting that people infected with one shared strain are more likely to be co-infected with other shared strains than unique strains. Alternatively, it can be argued that patients with advanced lung disease spending greater time in hospital are more likely to be exposed to one or more shared strains [18].

Single-strain infection with LES was previously shown to be associated with increased treatment requirements [27]. Our current study provides some evidence that infection with multiple shared strains is associated with greater morbidity than single-strain infection in Australia. In the year before recruitment and the months following their exacerbation episodes, those with mixed-strain infections had more treatment requirements than those with predominant single-strain infection, although this difference did not reach statistical significance. Further studies involving larger patient numbers and multiple centres are needed to confirm this observation. The data also potentially suggest that short-term intravenous antibiotic courses might not be a driver of the evolution of single strains dominating infection in CF patients. However, given that seven patients harboured a single-strain infection throughout the study period, longitudinal studies are required to determine if one shared strain displaces other(s) in mixed-strain infections as suggested by our observations and reported occasionally by others [15, 28, 29].

Patients with single-strain infections had a higher start-of-treatment CRP with improved lung function observed during treatment of the exacerbation. In contrast, although numbers were limited, those with mixed-strain infection had less evidence of an acute inflammatory response at the start-of-treatment and no significant change in lung function was observed. This further highlights the complexity of defining CF exacerbations and determining the timing and duration of antibiotic therapy, particularly in those with advanced lung disease, who now live longer than previously [30].

Although not statistically significant, we observed a transient decrease in the total *P. aeruginosa* load during the first 6-9 days of intravenous antibiotic treatment, which was reversed by the end of treatment. Similar transient effects have been previously described [31]. However, selection and clonal expansion of a *P. aeruginosa* population with a specific genotype or particular phenotypic traits may underpin pulmonary exacerbations [11, 15, 32, 33]. Whilst we cannot assign causality of exacerbation here, our in-depth sampling approach enabled the first characterisation of the relative abundance and dynamic nature of mixed strains, which cannot be determined if only a few isolates per sample are genotyped [13–15]. We found that the relative abundance of AUST-02 declined during anti-pseudomonal treatment [34]. At the same time, the relative abundance of other shared strains increased, suggesting they were under positive selection pressure. This was true especially for AUST-06, which was identified almost exclusively in Queensland previously and was the second most common shared strain identified here [13]. Whilst previous studies have shown within-host microevolution leads to co-existing sublineages of single *P. aeruginosa* strains emerging over months-to-years [26, 35–37], this study demonstrates rapid multi-strain turnover in mixed-strain infections within-host during antibiotic treatment of pulmonary exacerbations.

It is difficult to explain the observed temporal dynamics of mixed-strain infection, particularly when considering the antibiotic susceptibility profiles of strains from a single patient treated with meropenem, aztreonam and tobramycin. Even though the AUST-02 relative abundance decreased between start-of-treatment and end-of-treatment, this strain exhibited greater or equal in-vitro resistance to these antibiotics than the AUST-06 and AUST-07 strains that emerged in sputum during treatment. These unexpected results emphasise the poor correlation between in-vitro susceptibility testing and in-vivo response [38–40] and suggest some strains harbour alternative mechanisms, such as alginate overproduction and adaptive resistance that enable persistence despite aggressive antibiotic treatment of exacerbations [11, 41–45]. In addition, other virulence determinants may impact the host inflammatory response and co-infection with other CF pathogens [46–48]. Such factors could potentially favor the selection of AUST-06 during intravenous antibiotic treatment. Furthermore, phenotypic traits could generally be assigned to a particular strain type, but more isolates are required to confirm this observation and relate findings to the clinical course [11, 49].

While more than 2000 *P. aeruginosa* isolates were genotyped, the results must be interpreted with caution given the small number of patients attending a single centre involved. A further limitation is that sputum was not collected during clinical stability, before the onset of the exacerbation. Therefore, the proportions of strains at clinical stability before and after an exacerbation could not be compared. Sputum may also not be ideal for inferring the overall airway *P. aeruginosa* load or population composition as it may not represent all lung compartments, and whilst bronchoscopic sampling enables collection of regional *P. aeruginosa* populations, this method is too invasive for routine use [50]. Fluctuations in the population composition might represent regional changes in *P. aeruginosa* airway density and local micro-environmental conditions, altered mucus volume or variations in sputum sampling within the lung [37]. The patients had advanced lung disease and were treated with different antibiotic regimens, which may also affect the dynamics and evolution of mixed-strain infections. Furthermore, despite confirming AUST-02 by culture sweep at study entry, using a random culture approach, in two patients who had previously had chronic AUST-02 infection, AUST-02 was not subsequently identified in the genotyping of 48 randomly selected colonies, and only AUST-06 and AUST-01 were detected. It is possible AUST-02 might have constituted a minority of the *P. aeruginosa* population in these cases at the time of study recruitment, and therefore, were not selected because of limitations in sampling.

Further work will now be conducted to assess the relative abundance of shared strains directly in sputum using high-resolution molecular approaches and with larger study populations across a range of disease severities and other CF centres to validate the findings. This will extend to investigating intra-strain diversity and temporal dynamics, including an AUST-02 strain sub-type (M3 L7) that we described recently [51].

This exploratory study provides novel data characterising the temporal dynamics of a *P. aeruginosa* mixed-strain population during and after intravenous antibiotic treatment of exacerbations. Various commonly shared strains from throughout Australia, alone or in combination, were identified in individual patients. Together, the data show the rapidly changing strain heterogeneity of pulmonary exacerbations, raising further questions over whether acquiring shared *P. aeruginosa* strains is a marker or cause of more advanced CF lung disease. Ultimately, the much-needed answers to these questions will assist with refining treatments and existing infection control policies within CF centres.

## Conclusions

Within CF airways, multiple co-existing sublineages evolve gradually from a single dominant *P. aeruginosa* strain overtime. Combining in-depth sputum culture and genotyping techniques, we now also show that patients harbouring the AUST-02 strain may have other unrelated, but commonly shared, *P. aeruginosa* strains whose relative abundance with one another may change rapidly in a sustained and unpredictable manner during antibiotic treatment.

## Additional files

Additional file 1: Supplementary Methods. (DOCX 17 kb)

Additional file 2: Table S1. Characteristics of individual patients before the exacerbation episode. (DOCX 18 kb)

Additional file 3: Table S2. Treatment received during the exacerbation and clinical outcome data. (DOCX 18 kb)

Additional file 4: Figure S1. Timeline of sputa collection from 12 patients. (DOCX 48 kb)

Additional file 5: Table S3. Results of phenotypic testing of strains (AUST-02, AUST-06, AUST-07) isolated from each sputum sample provided by patient 1. (DOCX 27 kb)

Additional file 6: Figure S2. Temporal dynamics of the total proportions of AUST-02 and AUST-06 shared *Pseudomonas aeruginosa* strains detected during the course of intravenous antibiotic treatment of an exacerbation and subsequent follow-up for patients with mixed-strain infections. (DOCX 1092 kb)

Additional file 7: Figure S3. Characterization of adaptive phenotypic traits of within-patient (P1) mixed-strain infection. (DOCX 1101 kb)

## Abbreviations

ANOVA: Analysis of variance
BMI: Body mass index
CF: Cystic fibrosis
CFTR: Cystic fibrosis transmembrane conductance regulator
CFU: Colony-forming unit
CRP: C-reactive protein
FEV_1_%: Forced expiratory volume in the first second percentage predicted
PCR: Polymerase chain reaction
SNP: Single nucleotide polymorphism
