# Supplementary material for: Antibiotic perturbation of mixed-strain Pseudomonas aeruginosa infection in patients with cystic fibrosis
Source: BMC Pulm Med. 2017 Nov 2;17:138. doi: 10.1186/s12890-017-0482-7 (PMC5667482; doi:10.1186/s12890-017-0482-7)
Supplement: Supplementary file 1 — Supplementary Methods. (DOCX 17 kb) [file 12890_2017_482_MOESM1_ESM.docx]

**Additional file 1: Supplementary Methods**

**Allele-specific polymerase chain reaction (PCR) for AUST-02**

Each isolate initially underwent PCR screening for AUST-02 and non-AUST-02 *P. aeruginosa* strains using primers listed in Table 1. Briefly, these primers were used to distinguish a single nucleotide polymorphism (SNP) present on the *mexZ* gene that is associated with the AUST-02 strain. The reverse primer (Con_Reverse) targeted conserved sequences across all *P. aeruginosa* strains, whereas the forward primers were designed to detect AUST-02 (AUST-02_Forward) or Non-AUST-02 (Non-AUST-02_Forward).

**Table 1. Primers used to screen for AUST-02 and non-AUST-02
*P. aeruginosa* strains.**

| Primer name | Primer sequence (5’-3’) |
| --- | --- |
| AUST-02_Forward | GCGCCTTCGGCCAGACT |
| Non-AUST-02_Forward | GCGCCTTCGGCCAGACC |
| Con_Reverse | CTGGCGTTTTCGTCGGGTA |

Two reaction mixes were used. The AUST-02 reaction comprised the Con_Reverse and AUST-02_Forward primers, and the Non-AUST-02 reaction comprised the Con_Reverse and Non-AUST-02_Forward primers. Each reaction mix contained 10 µL SYBR PCR mix (Invitrogen), 0.5 µM of forward and reverse primers, 2 µL of heat-denatured isolate DNA (prepared as described previously^1^) and DNase-free water made up to a total volume of
20 µL. The PCR amplification was performed on a Rotor-Gene Q instrument (QIAGEN Pty Ltd) with the following conditions: an initial enzyme activation step at 95°C for 2-min, followed by 40 cycles of two-step cycling (denaturation at 95°C for 15-sec, and cycling at 60^o^C for 30-secs, with fluorescent signal acquired at the latter 60^o^C step). A reaction was classified as AUST-02 positive when the cycle threshold [Ct] value was <30 cycles in the AUST-02 reaction and was lower (by approximately 10 cycles) than the Ct value in the Non-AUST-02 reaction. A reaction was classified as Non-AUST-02 positive when the Ct value was <30 cycles in the Non-AUST-02 reaction and was lower (by approximately 10 cycles) than the Ct value in the AUST-02 reaction. Isolates that provided Ct values ≥30 cycles in both the AUST-02 and Non-AUST-02 reactions were classed as non-*P. aeruginosa* (in this study, *n*=27). Isolates that tested positive for a non-AUST-02 *P. aeruginosa* strain underwent Sequenom iPLEX SNP-based strain typing to determine the strain.^2^

**References**

1. Anuj SN, Whiley DM, Kidd TJ, et al. Identification of *Pseudomonas aeruginosa* by a duplex real-time polymerase chain reaction assay targeting the *ecfX* and the *gyrB* genes. *Diagn Microbiol Infect Dis.* 2009;63(2):127-31.

2. Syrmis MW, Kidd TJ, Moser RJ, et al. A comparison of two informative SNP-based strategies for typing *Pseudomonas aeruginosa* isolates from patients with cystic fibrosis. *BMC Infect Dis*. 2014;14:307.
