# Supplementary material for: Antibiotic perturbation of mixed-strain Pseudomonas aeruginosa infection in patients with cystic fibrosis
Source: BMC Pulm Med. 2017 Nov 2;17:138. doi: 10.1186/s12890-017-0482-7 (PMC5667482; doi:10.1186/s12890-017-0482-7)
Supplement: Supplementary file 2 — Characteristics of individual patients before the exacerbation episode. (DOCX 18 kb) [file 12890_2017_482_MOESM2_ESM.docx]

**Additional file 2: Table S1.** Characteristics of individual patients before the exacerbation episode.

| Patient^a^ | Age Range (years) | Gender | Genotype | Chronic Pa infection (years) | FEV_1_% predicted^b^ | BMI (kg/m^2^)^b^ | Inhaled antibiotics | | Oral azithromycin | Diabetes | Clinical care in previous year (number) | | |
| --- | --- | --- | --- | --- | --- | --- | --- | --- | --- | --- | --- | --- | --- |
|  |  |  |  |  |  |  | Colistin | Tobramycin |  |  | Admissions | Inpatient days | OPC visits |
| P1 | 36-40 | Male | P.Phe508del homozygous | >10 | 36 | 24.9 | No | Yes | Yes | Yes | 5 | 70 | 15 |
| P2 | 21-25 | Female | P.Phe508del homozygous | >10 | 42 | 17.4 | No | Yes | Yes | No | 4 | 102 | 16 |
| P3 | 41-45 | Male | P.Phe508del homozygous | >10 | 30 | 19.8 | Yes | Yes | Yes | No | 2 | 26 | 15 |
| P4 | 31-35 | Male | P.Phe508del homozygous | >10 | 56 | 19.5 | Yes | Yes | Yes | No | 1 | 10 | 18 |
| P5 | 21-25 | Male | P.Phe508del heterozygous | >10 | 66 | 21.3 | Yes | No | Yes | No | 5 | 64 | 20 |
| P6 | 31-35 | Male | P.Phe508del homozygous | >10 | 28 | 25.0 | Yes | Yes | Yes | No | 9 | 99 | 12 |
| P7 | 31-35 | Male | P.Phe508del heterozygous | >10 | 30 | 21.9 | No | No | Yes | No | 3 | 42 | 13 |
| P8 | 26-30 | Male | P.Phe508del heterozygous | >10 | 64 | 26.7 | No | Yes | Yes | No | 0 | 0 | 3 |
| P9 | 31-35 | Female | P.Phe508del homozygous | >10 | 49 | 19.8 | Yes | Yes | Yes | No | 2 | 22 | 15 |
| P10 | 51-55 | Male | P.Phe508del heterozygous | >10 | 35 | 35.5 | Yes | Yes | Yes | Yes | 2 | 26 | 5 |
| P11 | 16-20 | Male | P.Phe508del heterozygous | 5 | 83 | 27.3 | No | Yes | Yes | No | 0 | 0 | 3 |
| P12 | 21-25 | Female | P.Phe508del heterozygous | >10 | 30 | 15.2 | No | Yes | Yes | No | 6 | 88 | 27 |

*Abbreviations:* BMI, body mass index; FEV1% predicted, forced expiratory volume in the first second percentage predicted; OPC, outpatient clinic; Pa, *Pseudomonas aeruginosa*.

^a^No patients were diagnosed with CF-related liver disease.

^b^Best FEV_1_% predicted and BMI measurement within 12-months before the exacerbation episode.
