# Supplementary material for: Antibiotic perturbation of mixed-strain Pseudomonas aeruginosa infection in patients with cystic fibrosis
Source: BMC Pulm Med. 2017 Nov 2;17:138. doi: 10.1186/s12890-017-0482-7 (PMC5667482; doi:10.1186/s12890-017-0482-7)
Supplement: Supplementary file 3 — Treatment received during the exacerbation and clinical outcome data. (DOCX 18 kb) [file 12890_2017_482_MOESM3_ESM.docx]

**Additional file 3: Table S2** Treatment received during the exacerbation and clinical outcome data.

| Patient | Treatment  Regimen (duration; days) | Sputa provided; no. | CRP (mmol/L) | | FEV_1_% predicted | | BMI (kg/m^2^) | | Relapse | Time-to-next  exacerbation; days |
| --- | --- | --- | --- | --- | --- | --- | --- | --- | --- | --- |
|  |  |  | Start | End | Start | End | Start | End |  |  |
| P1 | *i.v.* Meropenem + Tobramycin (1-14); *i.v.* Aztreonam + Tobramycin (14-22) | 4 | 0.0 | 3.4 | 27.8 | 30.3 | 25.2 | 26.7 | No | 117 |
| P2 | *i.v.* Meropenem + Tobramycin (1-13) | 4 | 10.0 | 13.0 | 41.6 | 41.6 | 17.4 | 17.4 | Yes^c^ | 68 |
| P3 | *i.v.* Aztreonam + Tobramycin + *oral.* Doxycycline (1-14);  *i.v.* Ceftazidime +Tobramycin + *oral.* Doxycycline (14-25) | 4 | 14.0 | 13.0 | 28.1 | 31.2 | 19.4 | 19.8 | No | 154 |
| P4 | *i.v.* Meropenem + Tobramycin (1-11) | 4 | 3.7 | 2.0 | 45.7 | 60.2 | 19.2 | 19.4 | No | 145 |
| P5 | *i.v.* Ceftazidime +Tobramycin (1-16) | 4 | 19.0 | 19.0 | 63.1 | 65.5 | 21.2 | 20.4 | Yes^d^ | 44 |
| P6 | *i.v.* Piperacillin-tazobactam + Aztreonam+ Tobramycin (1-13) | 4 | 19.0 | 18.0 | 30.3 | 32.0 | 25.1 | 25.1 | No | 85 |
| P7 | *i.v.* Meropenem + Tobramycin (1-14) | 4 | 17.0 | 2.2 | 20.2 | 33.7 | 21.6 | 21.9 | No | 125 |
| P8^a^ | *i.v.* Ceftazidime +Tobramycin (1-7); *i.v.* Ceftazidime + Meropenem (7-10) | 2 | 6.5 | 5.7 | 50.9 | 62.5 | 25.1 | 26.1 | No | NA |
| P9 | *i.v.* Aztreonam + Tobramycin+ *oral.* Ciprofloxacin (1-18);  *i.v.* Meropenem + Aztreonam + Tobramycin + *oral.* Ciprofloxacin (18-24) | 4 | 87.0 | 3.0 | 50.3 | 57.5 | 19.6 | 19.3 | No | 381 |
| P10 | *i.v.* Piperacillin-tazobactam + Tobramycin (1-12) | 4 | 50.0 | 12.0 | 31.2 | 36.0 | 35.5 | 35.2 | No | 95 |
| P11^b^ | *i.v.* Ceftazidime +Tobramycin (1-10) | 3 | 16.0 | 0.0 | 84.0 | 99.8 | 25.4 | 25.1 | No | 289 |
| P12 | *i.v.* Ceftazidime + Meropenem + Tobramycin (1-21) | 4 | 42.0 | 7.3 | 31.0 | 35.8 | 15.6 | 15.7 | No | 39 |

*Abbreviations*: CRP, C-reactive protein; FEV_1_% predicted, forced expiratory volume in the first second percentage predicted; *i.v.*, intravenous; Start, start-of-treatment; End, end-of-treatment; NA, non-applicable (no recurrence of exacerbation; cut-off date 24^th^ May 2016).

^a^No end-of-treatment or follow-up sputum sample provided.

^b^No end-of-treatment sputum sample provided.

^c^Patient P2 was readmitted on the day of scheduled follow-up outpatient clinic visit.

^d^Patient P5 was readmitted on the day of outpatient clinic visit.
