# Supplementary material for: Antibiotic perturbation of mixed-strain Pseudomonas aeruginosa infection in patients with cystic fibrosis
Source: BMC Pulm Med. 2017 Nov 2;17:138. doi: 10.1186/s12890-017-0482-7 (PMC5667482; doi:10.1186/s12890-017-0482-7)
Supplement: Supplementary file 4 — Timeline of sputa collection from 12 patients. (DOCX 48 kb) [file 12890_2017_482_MOESM4_ESM.docx]

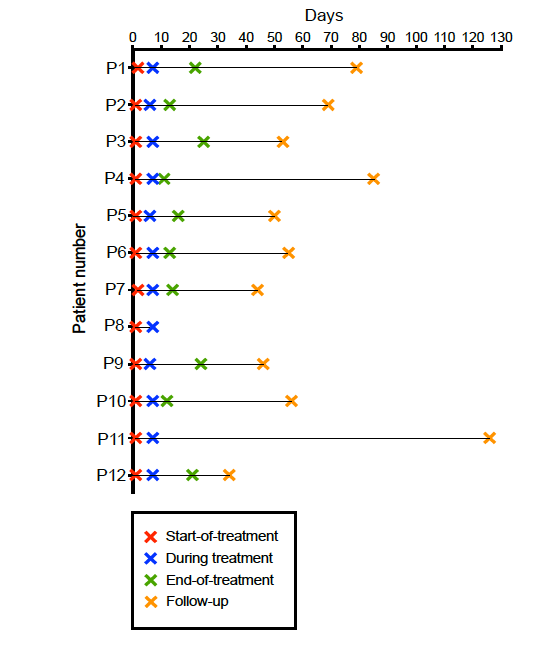


**Additional file 4: Figure S1**. Timeline of sputa collection from 12 patients. All patients provided a sputum sample at start-of-treatment (day 1) and during treatment (median, day 7; range, days 6-9). Ten patients provided an end-of-treatment sample (median, day 15; range, days 11-25) and 11 patients provided a follow-up sample (median, day 55; range, days 34-126).
