# Supplementary material for: Antibiotic perturbation of mixed-strain Pseudomonas aeruginosa infection in patients with cystic fibrosis
Source: BMC Pulm Med. 2017 Nov 2;17:138. doi: 10.1186/s12890-017-0482-7 (PMC5667482; doi:10.1186/s12890-017-0482-7)
Supplement: Supplementary file 5 — Results of phenotypic testing of strains (AUST-02, AUST-06, AUST-07) isolated from each sputum sample provided by patient 1. (DOCX 27 kb) [file 12890_2017_482_MOESM5_ESM.docx]

**Additional file 5: Table S3** Results of phenotypic testing of strains (AUST-02, AUST-06, AUST-07) isolated from each sputum sample provided by patient 1.

| Time-point  Antibiotic | Start-of-treatment; no. (%) | | | During treatment; no. (%) | | | End-of-treatment; no. (%) | | | Follow-up; no. (%) | | |
| --- | --- | --- | --- | --- | --- | --- | --- | --- | --- | --- | --- | --- |
|  | AUST-02 | AUST-06 | AUST-07 | AUST-02 | AUST-06 | AUST-07 | AUST-02 | AUST-06 | AUST-07 | AUST-02 | AUST-06 | AUST-07 |
| Aztreonam: R | 46 (100) | 1 (100) | - | 14 (93) | - | 2 (7) | 25 (96) | 20 (95) | 1 (100) | 10 (100) | 1 (100) | 11 (30) |
| I | 0 (0) | 0 (0) | - | 1 (7) | - | 4 (14) | 0 (0) | 0 (0) | 0 (0) | 0 (0) | 0 (0) | 7 (19) |
| S | 0 (0) | 0 (0) | - | 0 (0) | - | 22 (79) | 1 (4) | 1 (5) | 0 (0) | 0 (0) | 0 (0) | 19 (51) |
| Ceftazidime: R | 46 (100) | 1 (100) | - | 15 (100) | - | 4 (14) | 25 (96) | 11 (52) | 0 (0) | 10 (100) | 1 (100) | 11 (30) |
| I | 0 (0) | 0 (0) | - | 0 (0) | - | 2 (7) | 0 (0) | 0 (0) | 0 (0) | 0 (0) | 0 (0) | 3 (8) |
| S | 0 (0) | 0 (0) | - | 0 (0) | - | 22 (79) | 1 (4) | 10 (48) | 1 (100) | 0 (0) | 0 (0) | 23 (62) |
| Cefepime: R | 45 (98) | 1 (100) | - | 15 (100) | - | 2 (7) | 26 (100) | 20 (95) | 0 (0) | 9 (90) | 1 (100) | 9 (24) |
| I | 0 (0) | 0 (0) | - | 0 (0) | - | 3 (11) | 0 (0) | 0 (0) | 0 (0) | 0 (0) | 0 (0) | 3 (8) |
| S | 1 (2) | 0 (0) | - | 0 (0) | - | 23 (82) | 0 (0) | 1 (5) | 1 (100) | 1 (10) | 0 (0) | 25 (68) |
| Imipenem: R | 46 (100) | 1 (100) | - | 15 (100) | - | 28 (100) | 25 (96) | 20 (95) | 1 (100) | 10 (100) | 1 (100) | 35 (95) |
| I | 0 (0) | 0 (0) | - | 0 (0) | - | 0 (0) | 0 (0) | 0 (0) | 0 (0) | 0 (0) | 0 (0) | 0 (0) |
| S | 0 (0) | 0 (0) | - | 0 (0) | - | 0 (0) | 1 (4) | 1 (5) | 0 (0) | 0 (0) | 0 (0) | 2 (5) |
| Meropenem: R | 46 (100) | 1 (100) | - | 15 (100) | - | 9 (32) | 25 (96) | 19 (90) | 0 (0) | 10 (100) | 1 (100) | 23 (62) |
| I | 0 (0) | 0 (0) | - | 0 (0) | - | 9 (32) | 0 (0) | 0 (0) | 1 (100) | 0 (0) | 0 (0) | 6 (16) |
| S | 0 (0) | 0 (0) | - | 0 (0) | - | 10 (36) | 1 (4) | 2 (10) | 0 (0) | 0 (0) | 0 (0) | 8 (22) |
| Ticarcillin- R | 46 (100) | 1 (100) | - | 15 (100) | - | 14 (50) | 25 (96) | 18 (86) | 1 (100) | 10 (100) | 1 (100) | 25 (68) |
| clavulanate: I | 0 (0) | 0 (0) | - | 0 (0) | - | 2 (7) | 0 (0) | 0 (0) | 0 (0) | 0 (0) | 0 (0) | 6 (16) |
| S | 0 (0) | 0 (0) | - | 0 (0) | - | 12 (43) | 1 (4) | 3 (14) | 0 (0) | 0 (0) | 0 (0) | 6 (16) |
| Amikacin: R | 45 (98) | 1 (100) | - | 13 (87) | - | 10 (36) | 25 (96) | 2 (10) | 1 (100) | 9 (90) | 0 (0) | 17 (46) |
| I | 1 (2) | 0 (0) | - | 2 (13) | - | 8 (28) | 0 (0) | 0 (0) | 0 (0) | 1 (10) | 0 (0) | 5 (13.5) |
| S | 0 (0) | 0 (0) | - | 0 (0) | - | 10 (36) | 1 (4) | 19 (90) | 0 (0) | 0 (0) | 1 (100) | 15 (40.5) |
| Tobramycin: R | 5 (11) | 0 (0) | - | 6 (40) | - | 0 (0) | 11 (42) | 1 (5) | 0 (0) | 4 (40) | 0 (0) | 1 (3) |
| I | 7 (15) | 0 (0) | - | 0 (0) | - | 0 (0) | 3 (12) | 0 (0) | 0 (0) | 0 (0) | 0 (0) | 0 (0) |
| S | 34 (74) | 1 (100) | - | 9 (60) | - | 28 (100) | 12 (46) | 20 (95) | 1 (100) | 6 (50) | 1 (100) | 36 (97) |
| Ciprofloxacin: R | 4 (9) | 0 (0) | - | 3 (20) | - | 0 (0) | 6 (23) | 1 (5) | 1 (100) | 4 (40) | 0 (0) | 32 (87) |
| I | 10 (22) | 1 (100) | - | 6 (40) | - | 0 (0) | 10 (38.5) | 4 (19) | 0 (0) | 1 (10) | 0 (0) | 3 (8) |
| S | 32 (69) | 0 (0) | - | 6 (40) | - | 28 (100) | 10 (38.5) | 16 (76) | 0 (0) | 5 (50) | 1 (100) | 2 (5) |
| Polymyxin B: R | 1 (2) | 0 (0) | - | 0 (0) | - | 0 (0) | 0 (0) | 0 (0) | 0 (0) | 0 (0) | 0 (0) | 0 (0) |
| I | 0 (0) | 0 (0) | - | 0 (0) | - | 0 (0) | 0 (0) | 0 (0) | 0 (0) | 0 (0) | 0 (0) | 0 (0) |
| S | 45 (98) | 1 (100) | - | 15 (100) | - | 28 (100) | 26 (100) | 21 (100) | 1 (100) | 10 (100) | 1 (100) | 37 (100) |
| Colistin: R | 1 (2) | 0 (0) | - | 0 (0) | - | 0 (0) | 0 (0) | 0 (0) | 0 (0) | 0 (0) | 0 (0) | 0 (0) |
| I | 0 (0) | 0 (0) | - | 0 (0) | - | 0 (0) | 0 (0) | 0 (0) | 0 (0) | 0 (0) | 0 (0) | 0 (0) |
| S | 45 (98) | 1 (100) | - | 15 (100) | - | 28 (100) | 26 (100) | 21 (100) | 1 (100) | 10 (100) | 1 (100) | 37 (100) |
|  |  |  |  |  |  |  |  |  |  |  |  |  |
| Time-point  Phenotype | Start-of-treatment; no. (%) | | | During treatment; no. (%) | | | End-of-treatment; no. (%) | | | Follow-up; no. (%) | | |
|  | AUST-02 | AUST-06 | AUST-07 | AUST-02 | AUST-06 | AUST-07 | AUST-02 | AUST-06 | AUST-07 | AUST-02 | AUST-06 | AUST-07 |
| Auxotrophic: Yes | 44 (96) | 0 (0) | - | 11 (73) | - | 0 (0) | 25 (96) | 8 (38) | 0 (0) | 9 (90) | 0 (0) | 1 (3) |
| No | 2 (4) | 1 (100) | - | 4 (27) | - | 28 (100) | 1 (4) | 13 (62) | 1 (100) | 1 (10) | 1 (100) | 36 (97) |
| Iridescent sheen: Yes | 3 (7) | 0 (0) | - | 8 (53) | - | 26 (93) | 13 (50) | 0 (0) | 0 (0) | 6 (60) | 0 (0) | 31 (84) |
| No | 43 (93) | 1 (100) | - | 7 (47) | - | 2 (7) | 13 (50) | 21 (100) | 1 (100) | 4 (40) | 1 (100) | 6 (16) |
| Mucoidy: Yes | 0 (0) | 1 (100) | - | 0 (0) | - | 2 (7) | 1 (4) | 18 (86) | 0 (0) | 0 (0) | 1 (100) | 0 (0) |
| No | 46 (100) | 0 (0) | - | 15 (100) | - | 26 (93) | 25 (96) | 3 (14) | 1 (100) | 10 (100) | 0 (0) | 37 (100) |
| Pyocyanin Yes | 8 (17) | 0 (0) | - | 4 (27) | - | 4 (14) | 0 (0) | 0 (0) | 0 (0) | 1 (10) | 0 (0) | 5 (14) |
| production: No | 38 (83) | 1 (100) | - | 11 (73) | - | 24 (86) | 26 (100) | 21 (100) | 1 (100) | 9 (90) | 1 (100) | 32 (86) |

R, resistant; I, intermediate, S, susceptible; -, no strain isolated.
