# Supplementary material for: Antibiotic perturbation of mixed-strain Pseudomonas aeruginosa infection in patients with cystic fibrosis
Source: BMC Pulm Med. 2017 Nov 2;17:138. doi: 10.1186/s12890-017-0482-7 (PMC5667482; doi:10.1186/s12890-017-0482-7)
Supplement: Supplementary file 6 — Temporal dynamics of the total proportions of AUST-02 and AUST-06 shared Pseudomonas aeruginosa strains detected during the course of intravenous antibiotic treatment of an exacerbation and subsequent follow-up for patients with mixed-strain infections. (DOCX 1092 kb) [file 12890_2017_482_MOESM6_ESM.docx]

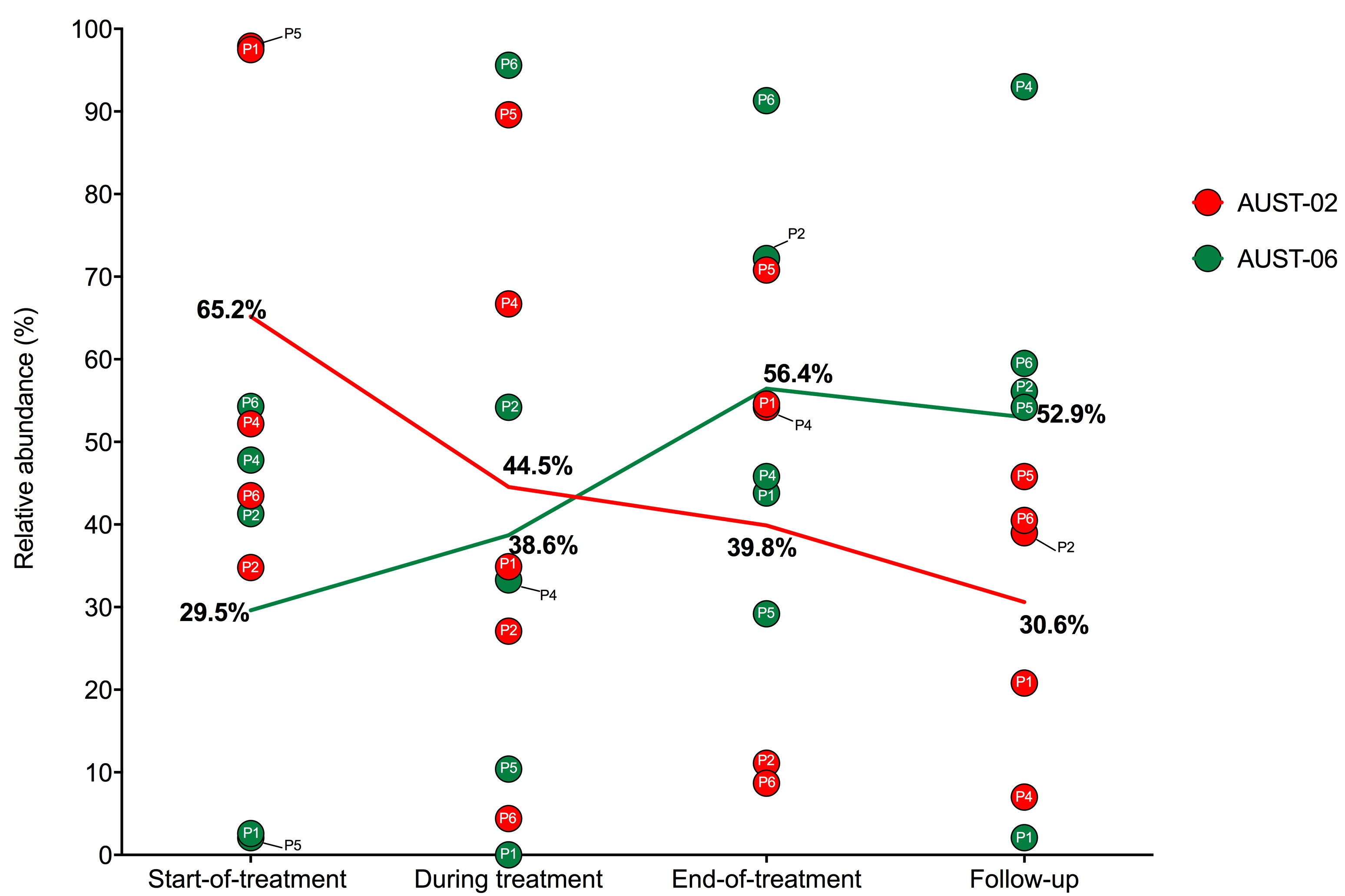


**Additional file 6: Figure S2** Temporal dynamics of the total proportions of AUST-02 and AUST-06 shared *Pseudomonas aeruginosa* strains detected during the course of intravenous antibiotic treatment of an exacerbation and subsequent follow-up for patients with mixed-strain infections. Each circle represents the relative abundance of AUST-02 (red) or AUST-06 (green) detected by random sampling within each person (*n*=5) and is based on genotyping results. The mean relative abundance is shown at each time-point. Percentages do not add up to 100% as some patients were also infected with AUST-07 or unique strains.
