# Supplementary material for: Antibiotic perturbation of mixed-strain Pseudomonas aeruginosa infection in patients with cystic fibrosis
Source: BMC Pulm Med. 2017 Nov 2;17:138. doi: 10.1186/s12890-017-0482-7 (PMC5667482; doi:10.1186/s12890-017-0482-7)
Supplement: Supplementary file 7 — Characterization of adaptive phenotypic traits of within-patient (P1) mixed-strain infection. (DOCX 1101 kb) [file 12890_2017_482_MOESM7_ESM.docx]

**
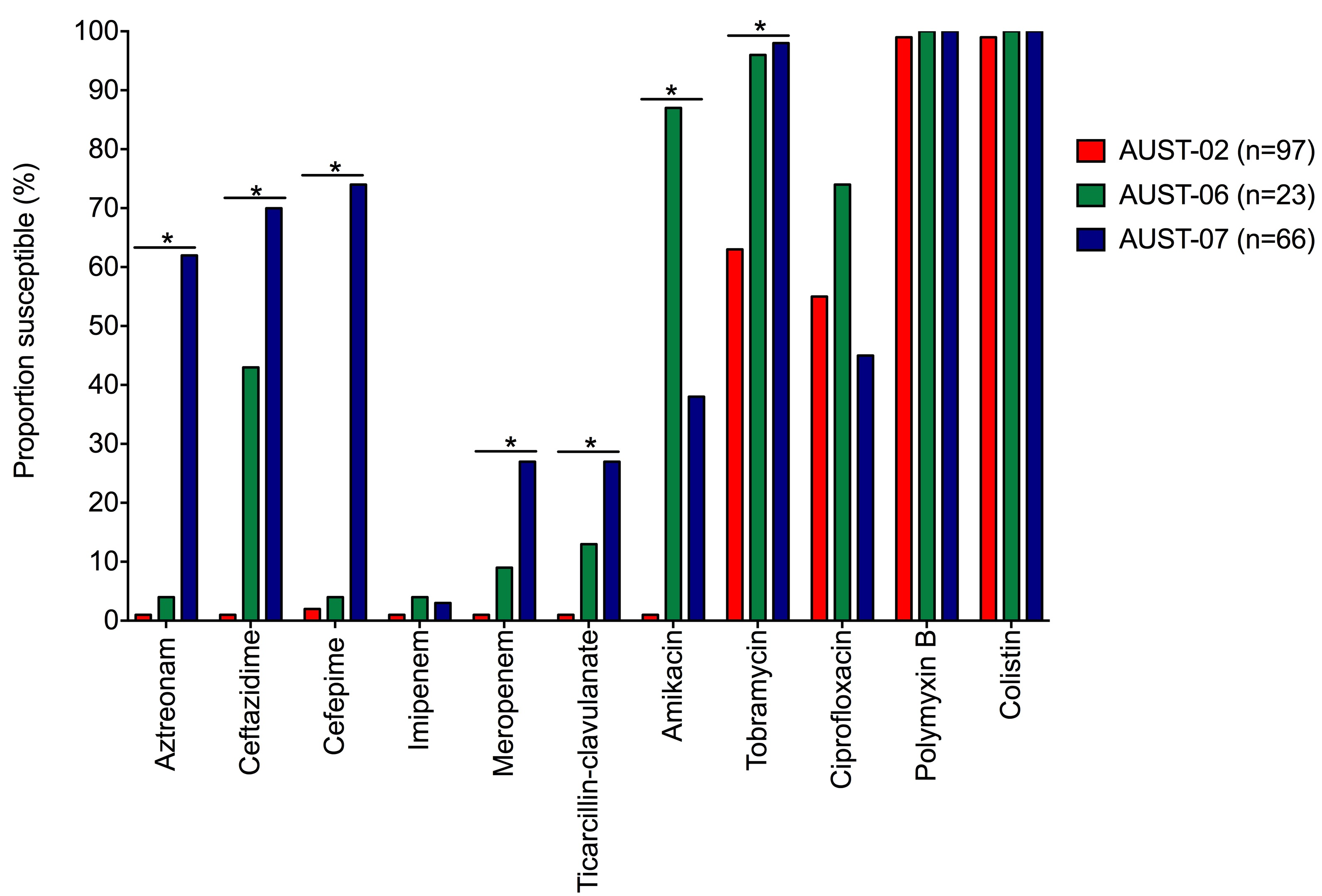
Additional file
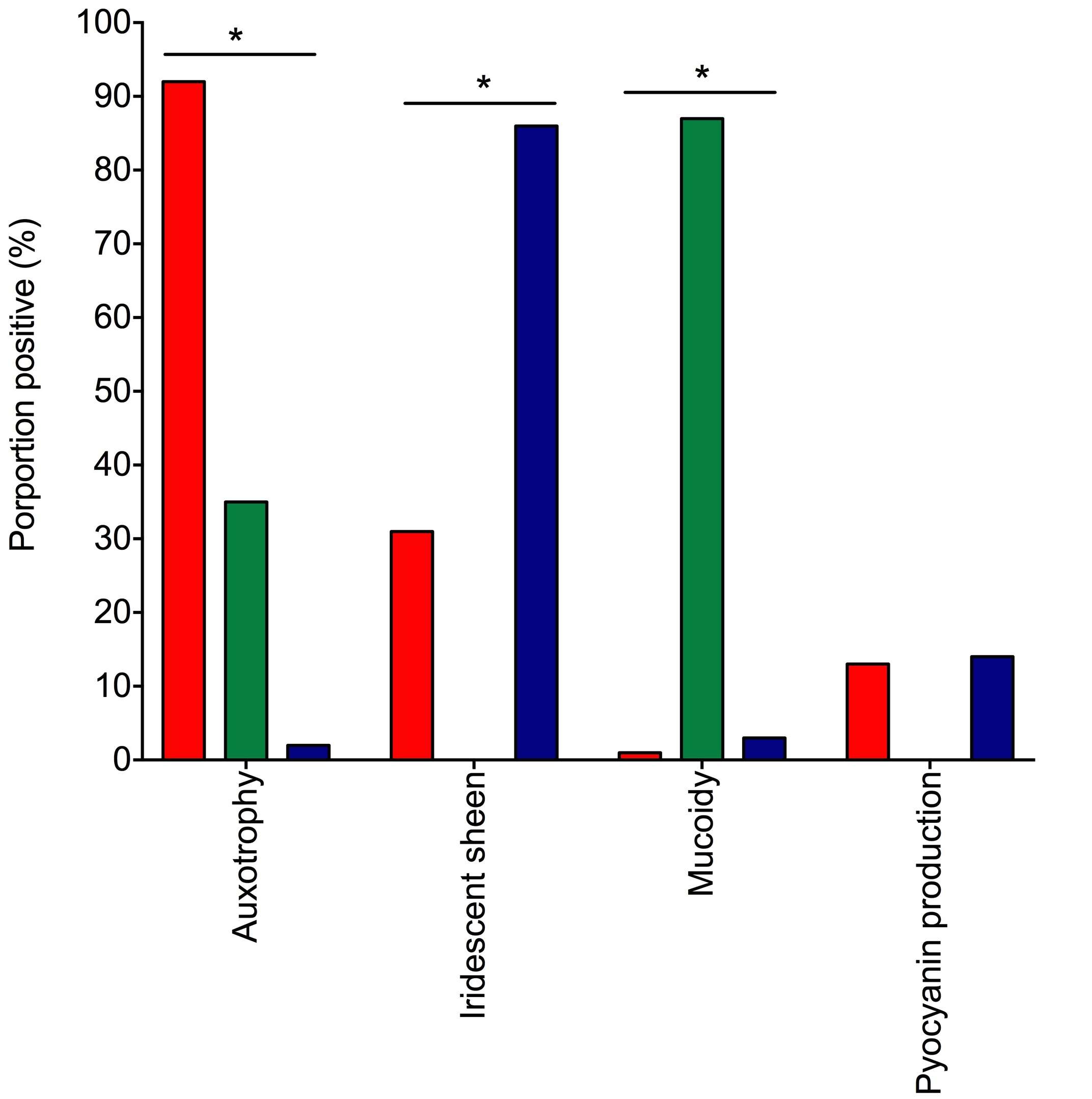
 7: Figure S3** Characterization of adaptive phenotypic traits of within-patient (P1) mixed-strain infection. AUST-02 (red bar, *n*=97);
AUST-06 (green bar, *n*=23); AUST-07 (blue bar, *n*=66). **P* <0.001.

(b)

(a)

(a) Percentage of strains categorized as susceptible to the antibiotics tested.

(b) Percentage of strains positive for auxotrophy, mucoidy, iridescent sheen (*lasR* mutants) and pyocyanin production.
